# Secondary Metabolites from *Croton* Species and Their Biological Activity on Cell Cycle Regulators

**DOI:** 10.3390/metabo15040216

**Published:** 2025-03-23

**Authors:** Jorge Augusto Alamillo-Vásquez, Claudia-Anahí Pérez-Torres, Enrique Ibarra-Laclette, Feliza Ramón-Farías, Pilar Nicasio-Torres, Fulgencio Alatorre-Cobos

**Affiliations:** 1 Red de Estudios Moleculares Avanzados (REMAV), Instituto de Ecología, A.C. (INECOL), Carretera Antigua a Coatepec 351, Col. El Haya, Xalapa 91073, Veracruz, Mexico; jorge.alamillo@posgrado.ecologia.edu.mx (J.A.A.-V.); enrique.ibarra@inecol.mx (E.I.-L.); 2 SECIHTI-Red de Estudios Moleculares Avanzados (REMAV), Instituto de Ecología, A.C. (INECOL), Carretera Antigua a Coatepec 351, Col. El Haya, Xalapa 91073, Veracruz, Mexico; 3 Facultad de Ciencias Biológicas y Agropecuarias, Universidad Veracruzana, Córdoba 94500, Veracruz, Mexico; felizarf@hotmail.com; 4 Centro de Investigación Biomédica del Sur (CIBIS), Instituto Mexicano del Seguro Social (IMSS), Xochitepec 62790, Morelos, Mexico; pisaliva@yahoo.com.mx; 5 SECIHTI-Unidad de Biología Integrativa Centro de Investigación Científica de Yucatán, A.C., Calle 43 No. 130 × 32 y 34, Col. Chuburná de Hidalgo, Mérida 97205, Yucatán, Mexico

**Keywords:** tropical plant species, plant metabolites, cell cycle, apoptosis, cancer

## Abstract

Plant-based traditional medicine integrates beliefs, knowledge, and practices to prevent and treat multiple diseases. *Croton* is a large and worldwide-spread genus belonging to Euphorbiaceae, a family well known for comprising many species with medicinal properties due to its high diversity of phytochemical constituents with biological activities. Among the various benefits of *Croton* species in traditional medicine, its use in cancer treatment has recently received significant attention from the scientific community. This review provides a general overview of different studies on the *Croton* genus in the research for alternative cancer treatments and the impact of its secondary metabolite catalog on cell cycle targets. Our analysis indicates that just under 30 secondary metabolites have been identified so far in latex and extracts obtained from leaves, twigs, or bark from 22 different *Croton* species. Based on standard assays using cell lines or human platelets, these molecules show multiple biological activities mainly compromising cell viability and cell cycle progression, supporting the ethnobotanical use of *Croton* species for cancer treatment. Several studies indicate that *Croton* metabolites target CDK–cyclin complexes and signaling routes that trigger apoptosis; however, further studies are needed to better understand the molecular mechanisms underlying *Croton* metabolites’ effects and their accurate future applications in cancer treatment.

## 1. Introduction

The Euphorbiaceae family comprises nearly 322 genera and 8910 species, and is one of the most diverse groups of angiosperms. For example, in Mexico, this botanic family includes 782 species grouped into 43 genera, some with highly contrasting phenotypes, for example, *Euphorbia*, *Croton*, and *Acalypha* [1]. Although this family contains the most famous flower globally, *Euphorbia pulcherrima* Willd. Ex Klotzsch, commonly known as poinsettia, is also appreciated for its secondary metabolites, which have biological activities against pathogens, insects, pests, and predators, and expanded use in traditional medicine for human wellness. They also have varied applications in the rubber industry (*Hevea brasiliensis* Müll.Arg.) and the food industry (*Manihot esculenta* Crantz).

**Figure 1:**
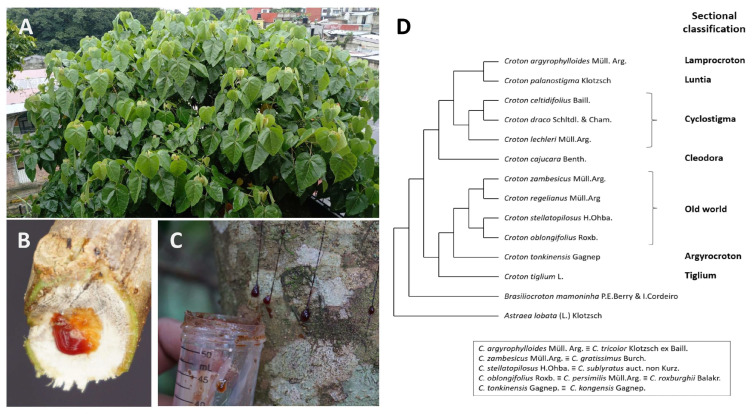
Some *Croton* species with ethnopharmacological studies associated with cell cycle regulators. (**A**) *C. draco* Schltdl var. draco tree growing in Mexico. In this species, secondary metabolite-rich latex can be extracted from (**B**) branches and (**C**) stems. (**D**) Sectional classification of *Croton* species with applications in cancer treatment.

*Croton* is one of the largest genera within the Euphorbiaceae family, containing about 1300 species with contrasting phenotypes ranging from large woody trees to herbs, and habits that include tropical and temperate zones worldwide [2]. Considering the complexity of the *Croton* genus, which has been demonstrated by both classical taxonomy studies and phylogenetic analyses [3,4], it is undeniable that future studies based on high-throughput genotyping will be necessary to accurately identify the high diversity in the *Croton* species [5]. Interestingly, two-thirds of all the *Croton* species described so far are distributed in the New World, particularly in South America [6]. For example, *C. lechleri* Müll.Arg., the most studied *Croton* species, is found in the Amazon region at an altitudinal range of 705–1660 asl. This species has been widely studied in pharmacology due to its importance in folk medicine; native people from this region treat several illnesses, tumors, and cancers with this natural resource [7]. In the Neotropic area, Mexico harbors over one hundred *Croton* species and is considered an essential center of secondary diversification of this genus [8]. For instance, *C. draco* Schltdl. var. draco has been documented for its ethnobotanical uses in this country (Figure 1A–C). This species is distributed from the Gulf of Mexico to the Yucatan Peninsula at altitudes that reach up to 1700 asl. In contrast to *C. lechleri*, *C. draco* has been poorly investigated despite their close taxonomic relationship (Figure 1B), opening a new research field on the phytochemistry and pharmacology of both species. Although the uses of *Croton* species are extended in traditional human medicine worldwide, their application in cancer treatment has recently received significant attention from the scientific community, especially those species with secondary metabolites (SMs) with bioactivity against cell cycle regulators [9] (Figure 1D). The main aim of this review is to provide a general overview of different studies on the *Croton* genus in research on alternative cancer treatments and the impact of its secondary metabolite catalog on cell cycle progression.

### Review Methodology

Relevant references were retrieved from PubMed, Web of Science, Springer, and Wiley Online Library databases from 1993 to 2025. The search terms included the words “*Croton*”, “anti-cancer/tumor”, “cell cycle”, “Tropical plant species”, “plant metabolites”, “apoptosis”, and “cancer combined with natural products”. The references in these articles were further checked to identify additional studies. Additionally, a brief search was carried out in Espacenet, Patentscope, and Google Patents using *Croton* as the keyword to identify developing novel inventions that meet patentability criteria in this topic.

## 2. Overview of Ethnopharmacological Uses of *Croton* Secondary Metabolites

Several *Croton* species produce red latex, commonly called “Dragon’s blood”. This crude extract is enriched with SMs such as taspine (the major component), tannins, diterpenes, and volatile oils [10]. *Croton* tree trunks, the preferential source of “Dragon’s blood”, contain laticifers and nonspecialized parenchyma cells that secrete latex in *Croton* species. The number of laticifers varies between species and depends on diverse factors such as plant age and environmental conditions [11,12]. *Croton* leaves are also an organ widely used in traditional medicine; however, it is not known what leaf tissues or cells produce the SMs that are potentially responsible for their biological activities.

**Figure 2:**
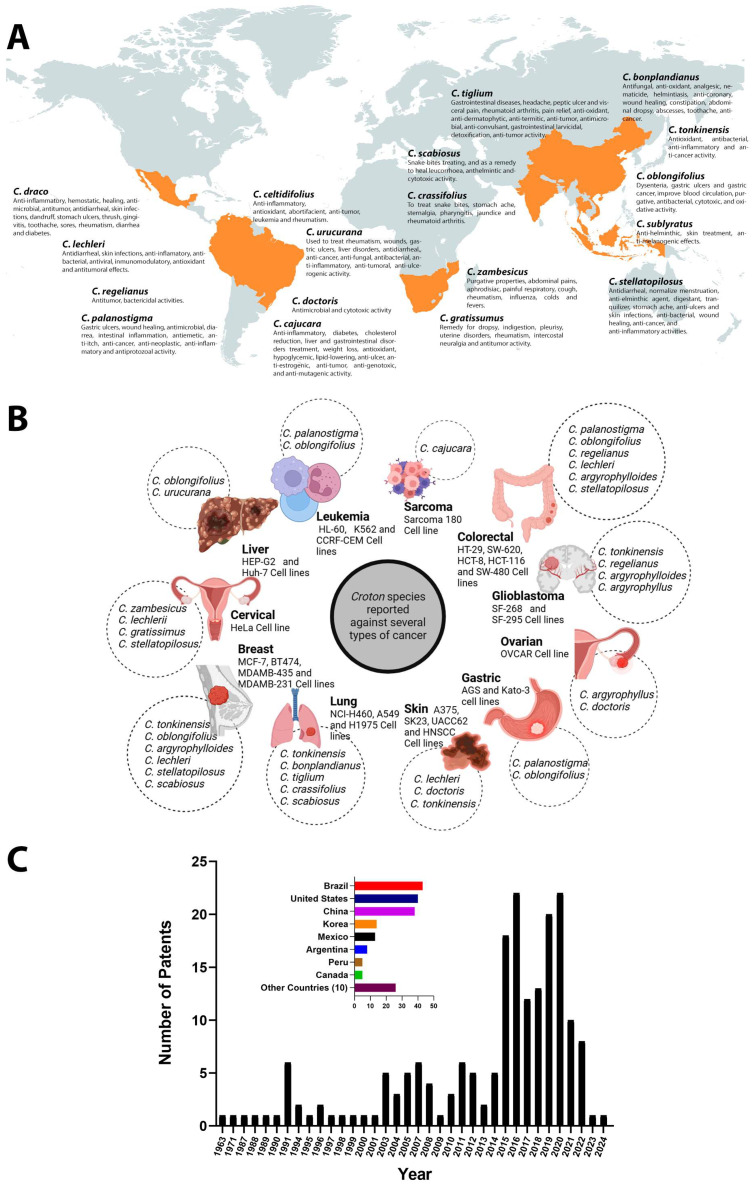
Traditional uses of and patents for *Croton* species worldwide. (**A**) Geographical distribution of scientific publications detailing ethnopharmacological applications by country. (**B**) Summary of cancer cell lines reported in *Croton* antiproliferative assays. (**C**) Patents protecting biotechnological and pharmacological uses of *Croton* species reported in the last 61 years. Diagrams created with BioRender.

Figure 2A shows the most common *Croton* species used in traditional medicine. These 18 species are distributed in Mexico, South America, Southern Africa, India, China, and Southeast Asia. In all of these countries, *Croton* species are an important resource for healing wounds [10,13,14]. They also act as anti-inflammatory agents [15,16,17] and treat respiratory and stomach illnesses [18], parasitic diseases [19,20,21,22], microbial infections [23,24,25,26], viral infections like hepatitis and herpes simplex [27,28,29,30], ulcers [31,32,33], diabetes [34,35,36,37], hypertension [38,39,40,41], rheumatic diseases [42,43], and cancer (Figure 2B) (for further studies focused on broader ethnobiological approaches and exploitation of scientific resources based on *Croton* species, we recommend other reviews available in the literature [10,44,45,46]). Because of the increasing impact on millions of human lives each year, research on *Croton* latex and the isolated SMs has focused on cancer, evaluating their effectiveness on cell proliferation and in vivo toxicity. The cancer types and cell lines evaluated include breast (MCF-7, BT474, MDAMB-435, and MDAMB-231), cervical (HeLa), gastric (AGS, Kato-3), colorectal (HT-29, SW-620, HCT-8, HCT-116, and SW-480), Leukemia (HL-60, K562, and CCRF-CEM), lung (NCI-H460, A549, and H1975), glioblastoma (SF-268 and SF-295), hepatocarcinoma (HEP-G2 and Huh-7), melanoma (SK23 and UACC62), and ovarian (OVCAR) (Figure 2B).

In line with the growing scientific interest in the *Croton* species, the number of patents for *Croton*-related use has also significantly increased in the last decade. Based on the search criteria, the results revealed approximately 20 patents per year in 2014–2020 compared with one per year in the 1990s. The patents protect protocols for fraction preparations, formulation methods, and use in areas such as pharmacology, cosmetics, and biotechnology. Interestingly, this emerging boom to protect the uses and innovations of *Croton* species is also occurring in countries that have long used *Croton* as an essential part of their traditional medicine (Figure 2C).

## 3. Molecular Targets of *Croton* Metabolites in Cancer

**Figure 3:**
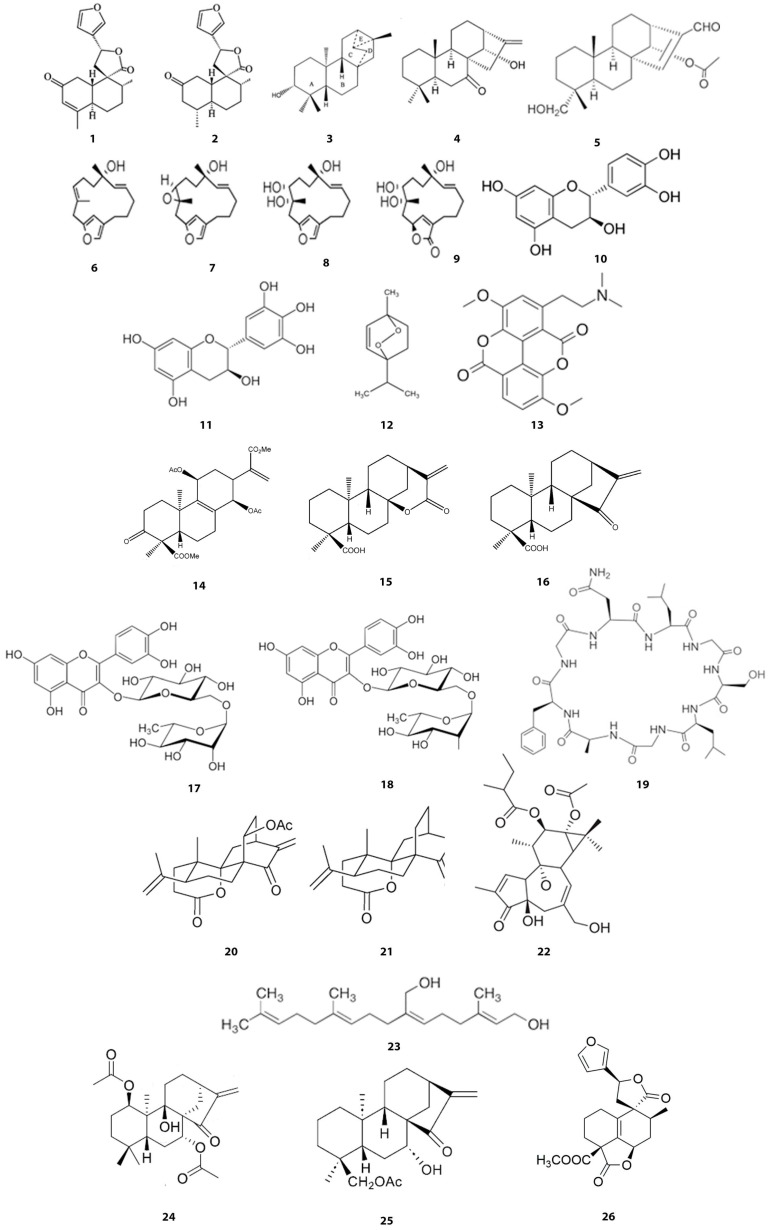
Chemical structures of SMs with reported cell cycle-related targets that have been identified in *Croton* species: (1) Trans-dehidrocrotonin, (2) Trans-crotonin, (3) *Ent*-trachyloban-3,-ol, (4) Crotonquinin A, (5) Crotonquinin B, (6–9) Furanocembranoids 1-4, (10) Catechin, (11) Galocatechin, (12) Ascaridol, (13) Taspine, (14–16) *Ent*-kaurene 1-3, (17) Rutin, (18) Vitexin, (19) Crourorb A1, (20) Crotobarin, (21) Crotogoudin, (22) B10G5, (23) Plaunotol, (24) *Ent*-kaurane I, (25) NCKU_PCKuo_0001, and (26) Chettaphanin II.

Cancer pathology is characterized by rapid cell proliferation beyond their boundaries; thus, this disease can invade other parts of the body, spreading malignancy caused by genetic alterations that modify cell cycle progression in different ways. Anticancer molecules approved for cancer therapy belong to natural plant products, such as irinotecan, vincristine, etoposide, camptothecin, and taxol [47]. As described above, *Croton* species are widely studied to determine their effects on several cancers. In at least 22 species, the biological activity observed has been associated with the crude extract (latex) or a specific isolated metabolite (Table 1, Figure 2 and Figure 3). Comprehension of the anticancer compounds’ activities and their molecular mechanisms of action is crucial to ensuring their safety in further clinical studies [48]. Several compounds found in *Croton* species have been evaluated for their role in fighting cancer by targeting cell cycle pathways (Table 1 and Figure 3). Most of the studies are focused on analyzing the role of these metabolites in cell cycle arrest and apoptotic pathways, with solid evidence in these processes. Following, we summarize what is known about these *Croton* SMs and the mechanisms involved in their use in cancer studies.

### Cell Cycle Arrest and Apoptotic Induction

The cell cycle represents a tightly programmed event that leads to a cell duplicating its content, mainly characterized by DNA replication and segregation of replicated chromosomes to generate two identical daughter cells. This process includes two principal differential stages: mitosis and interphase. Mitosis refers to the proper cell division that comprises prophase, metaphase, anaphase, and telophase. On the other hand, the interphase is the most extended phase of the cell cycle; during this time, the cell grows and replicates DNA in preparation for mitosis. The cell grows in size and volume before (G1 phase) and after (G2 phase) DNA synthesis (S phase). After cell division, some cells enter a non-replicative phase designated G0. This state can be temporary or definitive, as cells can rest in a cell cycle or be fully differentiated.

Cyclin-dependent kinases (CDKs), a family of serine/threonine protein kinases, control cell cycle progression in all eukaryotes. CDK activity is positively and negatively regulated by phosphorylation by protein subunits called cyclins, which bind to CDKs and generate a regulator complex. In addition to cyclins, other inhibitory and scaffolding proteins also join this complex to ensure specificity and subcellular localization. Because they control cell cycle progression, these key components are targets in the search for cancer therapies since their expression in cancer cells is compromised; therefore, their regulation could induce cell death [78]. Progression through G1/S is led by the CDK–cyclin complex that regulates the retinoblastoma protein/E2F (RB/E2F) pathway, which is crucial in regulating cell cycle gene expression. Increased intracellular levels of cyclin D lead to the formation of CDK4–cyclin D and CDK4–cyclin D complexes, which phosphorylate the retinoblastoma protein (RB) [79]. The activity of the E2F transcription factor, whose products are required for S phase progression, is repressed by RB. To progress through G1/S, hyperphosphorylation of RB protein by the action of the CDK2–cyclin E complex has to take place in late G1. Therefore, RB/E2F members are cell cycle regulators. Beyond this restriction point, the CDK1–cyclin A complex keeps RB phosphorylated, ensuring the S phase. Genetic alterations of molecular determinants involved in the G1-S transition are present in different cancer types, allowing cells to proliferate independently. Therefore, discovering and studying new inhibitors of cell regulators such as CDKs is a hot point in the search for anticancer therapeutics [80].

*Croton* latex and isolated SMs have been evaluated to assess their in vitro effectiveness against the proliferation of cell lines, identifying molecular targets throughout cell cycle progression. In the initial stages of the cell cycle, B10G5, a naturally occurring phorbol ester isolated from *C. tiglium* (Figure 3), increases apoptosis and leads to decreased levels of cyclin D1 in the NSCLC lung cancer cell line [71]. As cyclin D1 is required for G1/S progression and cyclin D overexpression is associated with tumor growth [81], B10G5 represents a promising SM for developing a specific cancer therapy targeting this phase.

In addition to modulating cyclin D1 levels, B10G5 activates the protein kinase C (PKC) pathway [71]. The activities of the genes involved in this pathway are generally compromised in many types of cancers, usually due to the suppression of their expression levels [82]. B10G5 affects the anti-proliferation of lung tissue cells, including apoptosis and cell cycle arrest in the G2/M phase, by activating PKC-mediated signaling pathways and downregulating cyclin B1, matching PKC roles in cellular functions such as proliferation, G2/M activity, and cyclin B1 modulation [83].

**Figure 4:**
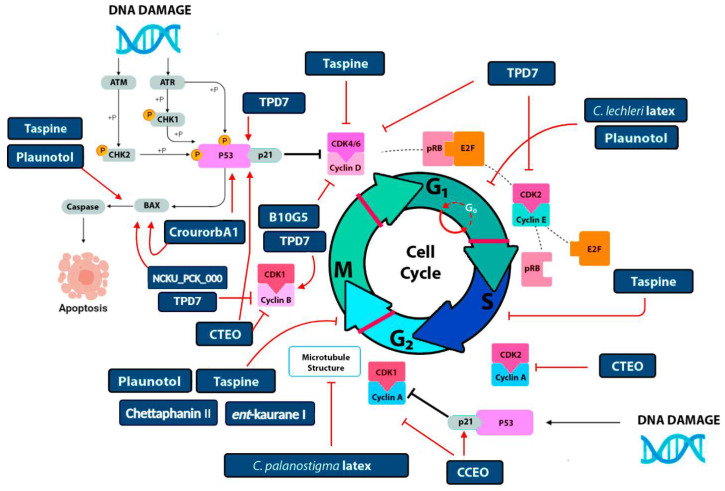
Schematic representation of cell cycle control by natural compounds from the *Croton* genus. Progression through the cell cycle involves the formation, activation, and successive participation of cyclin-dependent protein kinases (CDKs), which bind sequentially to a series of cyclins and regulate the cell cycle at different stages. Crude extracts and natural compounds isolated from the *Croton* genus, e.g., taspine, plaunotol, B10G5, chettaphanin II, NCKU_PCK_000, and *ent*-kaurane-I, have been shown to influence specific targets at different points, altering cell cycle progression through inhibition of CDK–cyclin complexes and overexpression of apoptosis mediators. In addition to cell cycle arrest induction, upstream signaling routes that sense DNA damage and trigger cell death programs via gene expression modulation have also been linked to *Croton* extracts such as CCEO, CTEO, TPD7, and crourorbA1. Diagram created with BioRender.

Taspine, an alkaloid first isolated from *Radix et Rhizoma* Leonticis, has become an important natural resource due to its extensive pharmacological activities. Interestingly, taspine is a major constituent of latex in some *Croton* species, representing up to 7–9% of latex dry weight (Figure 3) [84,85]. This alkaloid directly affects CDK2 and CDK4, key players in the early stages of the cell cycle. Treating the A431 cell line, a clinical model of epidermal carcinoma, with taspine metabolite altered CDK2/CDK4 levels and compromised G1/S transition, leading to BCL-2 downregulation and causing apoptosis and cell cycle arrest at G1/S transition [86] (Figure 4). BCL-2 family proteins play a pivotal role in regulating cell apoptosis due to their ability to form membrane channels in mitochondria. The proteins can be classified into two subfamilies: pro-apoptotic members (BCL-2, MCL-1) and anti-apoptotic members (BAX, BAK, BAD) [87]. Localized to the outer membrane of mitochondria, BCL-2 has a significant impact on mitochondrial integrity. Thus, *Croton* taspine could positively affect cancer treatment by affecting cell proliferation and apoptosis. In line with this idea, it has been proposed that apoptosis induced by *Croton* taspine is caused by the expression of specific genes with action patterns similar to topoisomerase inhibitors such as ellipticine and camptothecin [54]. Topoisomerases are enzymes involved in DNA supercoiling, a key point for cell replication. Indeed, taspine induces an accumulation of cells undergoing S and G2 phases of the cell cycle, similar to what topoisomerase inhibitors do (Figure 4).

Due to the importance of taspine demonstrated in recent years, attempts to improve solubility have led to a new taspine derivative, TPD7 [88], which has antiproliferative activity in cutaneous T cell lymphoma H9. TPD7 significantly decreased the levels of the anti-apoptotic BCL-2 and MCL-1 proteins and increased the levels of the pro-apoptotic BAX, BAK, and BAD proteins and, interestingly, P53, a tumor suppressor gene in H9 cells. Thus, the effect of TPD7 in inhibiting H9 cell viability appears to be mediated by induction of cell cycle arrest and increased cell apoptosis through the mitochondrial pathway. Additionally, TPD7 targets cell cycle regulators, such as CDC2 (CDK1), cyclin D1, and cyclin E1, which are essential in the G1/S transition, and cyclin B1, which is associated with the G2/M checkpoint. Although further research is required, these results show that *Croton* taspine and TPD7 provide insights for regulating cell cycle regulators (Figure 4), a key aspect during cancer treatment.

Further assays using confocal microscopy demonstrated that taspine increased acetylated α-tubulin, which is a marker of stable, long-lived, nondynamic microtubules [89,90,91] that trigger morphologic changes in the human melanoma SK23 cell line, suggesting its molecular action on specific targets in the cytoskeleton (Figure 4).

The diterpenoid *ent*-kaurane I, isolated from the twigs and leaves of *C. kongensis*, was evaluated for its cytotoxic activity against five human tumor cell lines. Biological tests showed that this compound possesses strong anti-proliferation activity, arrests the cell cycle at the G2/M phase, and induces apoptosis of MDA-MB-231 breast cancer cells [77]. Mitochondria may produce more ROS because mitochondrial function is weakened by MMP depolarization, and the excessive ROS may be involved in the regulation of cell apoptosis. Investigation of its mechanism showed that *ent*-kaurane I can inhibit tumor proliferation and migration by targeting mitochondria to increase intracellular reactive oxygen species (ROS) and regulating STAT3 and FAK signal pathways. Collectively, these findings support the great potential of this compound as an anticancer agent [77].

Natural antioxidants, such as phenols, tannins, and flavonoids, in the acetonic fraction of *C. bonplandianus* leaf extracts (AcE), exhibit anticancer activity against lung tumor cell lines. Treatment with AcE leads to cell cycle arrest in G2/M and cell death induction. Although further experimental research is needed to understand the molecular mechanisms, attempts to scavenge free radicals—commonly associated with the process and progression of cancer—using natural antioxidant sources could be an alternative strategy for treating cancer [65]. Another *Croton* metabolite evaluated in cancer research is plaunotol, a diterpene isolated and found only in *C. stellatopilosus* [70]. It acts as an apoptotic agent in breast, cervical, and colon cancers. Plaunotol exhibited antiproliferative activity during the G0/G1 phase in HeLa cells, while in the HT-29 line, a similar effect was observed in G2/M, leading to apoptosis in both cases (Figure 4). Expression profiling analysis showed that this *Croton* metabolite induced BCL-2 downregulation, similar to that previously found for taspine, which may explain cell death through mitochondrial-dependent pathways observed in the tested cell lines.

Another SM that induces G2 arrest and prevents mitotic entry is [1–9-NαC]-crourorb A1 [67], a cyclic peptide isolated from *C. urucurana.* The viability of Huh-7 (human hepatocarcinoma) cells treated with this peptide was compromised in a dose- and time-dependent manner, and was associated with the induction of apoptosis. The effects of crourorb A1 associated with G2/M arrest were due to changes in CDK1, cyclin B1, and P53 expression. Crucial checkpoints comprise activation of tumor suppressor gene P53 guided by DNA damage transducer kinases—ataxia telangiectasia mutated (ATM), Ataxia telangiectasia/Rad3 related (ATR), and checkpoint kinases CHK1 and CHK2—preventing CDK1–cyclin B activation and mitotic entry. P53 and G2/M modulation by [1–9-NαC]-crourorb A1 highlights the importance of the biological activity of natural cyclic peptides from *Croton* species in cancer cell lines [67].

Another report focused on the inhibition of oral squamous carcinoma cells (HNSCCs) and their radiosensitization by the NCKU_PCK_0001 compound isolated from *C. tonkinensis* [58]. This compound reduced cell viability, migration, and invasion by downregulating the AKT/mTOR signaling pathway and its mediators, such as pAKT (ser473), p-mTOR (ser2448), and PI3K, a heterodimer comprising two distinct subunits: the regulatory subunit p85 and the catalytic subunit p110α. Dysregulation of these proteins drives cancer development and progression [92,93,94]. Blocking this pathway modulates checkpoint kinase 2 (CHK2) activity and the resulting effects on (CDK1)/cyclin B1 activity as its potential mechanism on cell cycle [95]; thus, cell cycle arrest is the major mechanism of radiation sensitization associated with PI3K/mTOR inhibition by NCKU_PCK_0001 in HNSCCs. This suggests that compounds derived from *C. tonkinensis* could potentially improve the effectiveness of radiotherapy, especially in overcoming radioresistance, which is a common challenge in cancer treatment [58].

Natural *Croton* oils also potentially affect the development of cancer treatments; for instance, *C. crassifolius* Geisel essential oil (CCEO) induces cell cycle arrest in A549 lung tumor cells. The molecular targets of CCEO are cyclin B1-CDK1 and cyclin A-CDK1. CCEO acts by decreasing their expression levels and, in contrast, increasing cyclin-dependent kinase inhibitor (CKI) P21 expression [73]. Similarly, treating the A549 lung cancer cell line with *C. tiglium* essential oil (CTEO) altered the expression levels of cyclin B1, cyclin A, and CDK1 [72]. This triggered cell growth inhibition, with significantly enhanced P21 levels. P21 inhibits the activities of CDK2 and CDK4 in a P53-dependent or independent manner. Lastly, P53 can act as an oncogene, promoting tumor evolution by accumulating DNA damage. Determining the correct role of P21 in preventing cancer progression has been challenging in cancer research [96].

Chettaphanin II, isolated from *C. crassifolius* root extracts, recently showed a reduction in A549 human lung carcinoma cell proliferation and induced G2/M phase arrest, resulting in cytotoxicity and apoptosis [74]. Chettaphanin II significantly inhibited tumor growth in experiments performed on naked mice. The antitumor mechanism of chettaphanin II involves blocking the mTOR/PI3K/Akt signaling pathway in the A549 cells. Molecular docking established that chettaphanin II can bind to the active sites of BCL-2 and BAX. This report confirmed that chettaphanin II has strong tumor inhibition ability in vitro and in vivo, indicating its potential application in anticancer therapy [74].

The centrosome cycle occurs parallel to the cell cycle and is regulated by mitotic kinases such as CDK1 through spindle apparatus formation. During G2/M, the CDK1–cyclin B1 complex is activated to initiate M through chromosome condensation and microtubule dynamics regulation. To ensure the integrity of the genetic material at the end of the cell cycle, there are checkpoints to repair DNA damage that commonly occurs during the process and then activate cell arrest [97]. At this cell cycle point, one of the proposed mechanisms underlying apoptosis induced by *C. palanostigma* latex treatment is perturbations in microtubule structure adhesion and reassembly. Studies have reported that cancer cells undergo blockage upon exposure to latex during the late G2 phase of the cell cycle. Characteristic apoptotic changes, such as chromatin condensation and nucleus contraction, were evident within a few hours of treatment, resulting in non-functional microtubules being unable to adhere and leading to cell apoptosis [51].

## 4. Perspectives and Conclusions

The vast chemical diversity of the *Croton* genus is comparable with the number of its species, which represents a unique opportunity in the quest for novel anticancer agents. Mining the current literature during the writing of this review indicated that less than 5% of the known *Croton* species have been included in bioprospection metabolite studies. The pharmaceutical potential of the *Croton* genus is particularly significant for countries rich in these species; this is reflected in the increase in *Croton*-use-related patents claimed by countries harboring *Croton* species.

*Croton* latex and isolated SMs have been evaluated to assess their in vitro effectiveness against proliferation in cell lines and in vivo toxicity. Cancerous cells are well known for their capacity to maintain proliferative signaling, mainly via mutations that prevent apoptosis and cell cycle exit. Understanding how malignant cell machinery acts can unveil susceptible steps that could be targeted for new drug development. Compelling evidence collected so far shows that several *Croton* SMs exert biological activity against diverse and specific targets on cell cycle progression and signaling pathways triggering apoptosis; however, other molecular mechanisms or metabolic processes underlying their anticancer properties are still missing. For example, the role of phenolic compounds, enriched in the *Croton* latex, on redox homeostasis disruption or glucose transporters (GLUCTs) in cancerous cells is almost unknown. Epigallocatechin gallate from green tea is reported to decrease GLUT1 mRNA levels in colon cancer cells; gallocatechin has been found in *C. celtidifolius*; thus, a link between *Croton* SMs and GLUCTs is plausible. Other questions remain open about compounds like crotobarin, crotogoudin, crotonkinins, or the flavonoids rutin and vitexin, already reported in *Croton* species. The last flavonoid, vitexin, is a potential drug for nasopharyngeal carcinoma. Cell energy metabolism could be another hot research topic in *Croton*, especially in the mitochondrion, as reprogramming energy metabolism is one of the hallmarks of cancer therapy. Effects of *Croton* compounds on tubulin and actin have also been reported; regulation of the cytoskeleton plays a key role in cancer cell migration and invasion; thus, research in this field could provide insights into *Croton* SMs’ benefits in controlling cancer.

Given the significant heterogeneity among cancer types, even within the same tissue, it is imperative to understand in depth the mechanisms of action of each metabolite, including their specific molecular targets within the cell cycle and other metabolic processes. By addressing these challenges, the lack of insight into the clinical stages of *Croton* SMs can be solved, impacting the development of cancer precision medicine.

## Figures and Tables

**Table 1:** Compounds in the *Croton* genus that are involved in cell cycle processes and apoptosis. The extraction methods, doses, and experimental models are indicated.

Species	Molecule/Extraction Method/Dose	Study Model	Biological Activity/Target Regulation	Ref.
*C. cajucara*	DCTN and CTN/Hexane and Methanol/80 and 120 mg/Kg	Sarcoma-180 cells and Ehrlich carcinoma	Tumor inhibition, TNF-α	[49]
	*t*-DCTN/-/180 µM	HL-60 cells	Cytotoxic activity	[50]
*C. palanostigma*	Latex/Aqueous extract/100 µL/mL	HT-29, and HT-84 cells	Apoptosis, β-tubulin	[51]
*C. lechleri*	Latex/DMSO/2.5 µg/mL	K562 cells	Cellular proliferation inhibition	[52]
	Latex and taspine/Dichloromethane–Methanol/1 to 10 µg/mL	SK23 cells	Apoptotic activity α-tubulin, F-actin	[53]
	Taspine/-/10 mg/Kg	HCT-116 model	Apoptosis in vivo, Topoisomerase I, and II inhibitor	[54]
	Rutin and vitexin/Methanol/17 µg/mL	HeLa, SW-480, and MDAMB-231 cells	Apoptotic activity	[7]
	Twig extracts/Soxhlet ethanol and pressurized ethanol/13.31 to 249.8 µg/mL	HaCat and A375 cells	Apoptotic activity Cell cycle arrest	[55]
*C. zambesicus*	*Ent*-18 hidroxy-traquilobano-3β-ol/Dichloromethane extract/7.3 µg/mL	HeLa and HL-60 cells	Caspase-3/CPP32	[56]
*C. tonkinensis*	Crotonkinins A-B/Methanolic extract/0.61 to 1.45 µg/mL	MCF-7, A549, and KB cells	Cellular proliferation inhibition	[57]
	NCKU_PCKuo_0001/-/10 µM	HNSCC cell line	Cell viability and AKT/mTOR downregulation	[58]
*C. oblongifolius*	Furanocembranoids/Hexane fraction/5.6 to 9.5 µ g/mL	BT474, HEP-G2, Kato-3, and SW-620 cells	Cytotoxic activity	[59]
*C. celtidifolius*	Catequin and gallocatequin/Aqueous ethanol extract/200 µg/mL	Human platelets	Platelet aggregation inhibition	[60]
*C. regelianus*	Ascaridole and essential oils/Hidrodistilled extract/6.3 to 18.4 µg/mL	HL-60, SF-295, and HCT-8 cells	Cytotoxic activity	[61]
*C. argyrophylloides*	*Ent*-kaurene 1-3/Ethanol extract/1.5 to 8.2 µg/mL	HL-60, MDAMB-435, SF-295, and HCT-8 cells	Cytotoxic activity	[62]
*C. barorum*	Crotobarin and crotogoudin/Ethyl acetate extract/4 µM	Murine P388 cells	Cytotoxic activity Cell cycle arrest (G2/M)	[63]
*C. sylvaticus*	Root bark/Dichloromethane–Methanol/10 µg/mL	CCRF-CEM cells	Cytotoxic activity	[64]
*C. bonplandianus*	Leaf extract/Acetone extract/15.68 µg/mL	A549 cells	Cytotoxic and apoptotic activities	[65]
	Leaf extract/Ethanolic fraction/86.33 µg/mL	K562 cells	Cytotoxic activity	[66]
*C. urucurana*	Crourorb A1/Ethyl acetate/35.75 µg/mL	Huh-7 cells	CDK1, Cyclin B1, MAP Kinases JNK signaling	[67]
*C. gratissimus*	Leaf extracts/Acetone and ethanol/152.3 to 462.88 µg/mL	HeLa Cells	Cytotoxic activity and Caspase-3 and -7 activation	[68]
*C. argyrophyllus*	Essential oil/Hydrodistilled extract/14.81 to 32.79 µg/mL	MDAMB-435 and OVCAR cells	Cytotoxic activity	[69]
*C. stellatopilosus*	Plaunotol/Hexane extract/65.47 to 80.90 µg/mL	MCF7, KB, HeLa, and HT-29 cells	TNF-α, BCL-2, BAK/BAX	[70]
*C. tiglium*	B10G5/-/0.11 to 20 µM	A549 and H1975 cells	PKC activation, Cyclin B1, Cyclin D1, PARP	[71]
	CTEO/Supercritical CO_2_ fluid extract/48.38 µg/mL	A549 cells	Proliferation inhibition, cell cycle arrest. Cyclin and CDK inhibition.	[72]
*C. crassifolius*	CCEO/Supercritical CO_2_ fluid extract/25 µg/mL	A549 cells	Apoptosis, Cyclin B1, Cyclin A, CDK1	[73]
	Chettaphanin II/Supercritical CO_2_ fluid extract/8.58 µM	A549 cells	BAX, BCL-2, Cyt-C, Cell cycle arrest, mTOR/PI3K/Akt signaling	[74]
*C. doctoris*	Essential oils/Hexane fraction/13.4–21.8 µg/mL	UACC62 and OVCAR cells	Cytotoxic activity	[75]
*C. scabiosus*	Bark extracts/Chloroform and ethyl acetate/187.33 to 201.89 µg/mL	A549 and MCF7 cells	Cytotoxic activity	[76]
*C. kongensis*	*Ent*-kaurane I/Ethyl acetate extract/1 to 4 µM	MDA-MB-231 cells	Apoptotic activity and regulation of STAT3 and FAK signal pathways	[77]

## Data Availability

No new data were created or analyzed in this study. Data sharing is not applicable to this article.

## Abbreviations

The following abbreviations are used in this manuscript:

CDK	Cyclin-dependent Kinase
SMs	Secondary metabolites
RB	Retinoblastoma protein
